# Antenatal non-medical risk assessment and care pathways to improve pregnancy outcomes: a cluster randomised controlled trial

**DOI:** 10.1007/s10654-018-0387-7

**Published:** 2018-03-31

**Authors:** Jacqueline Lagendijk, Amber A. Vos, Loes C. M. Bertens, Semiha Denktas, Gouke J. Bonsel, Ewout W. Steyerberg, Jasper V. Been, Eric A. P. Steegers

**Affiliations:** 1 Department of Obstetrics and Gynaecology, Erasmus MC, University Medical Centre, PO Box 2040, 3000 CA Rotterdam, The Netherlands; 2 Department of Social and Behavioural Sciences, Erasmus University College, Faculty of Social & Behavioural Sciences, Erasmus University Rotterdam, Nieuwemarkt 1A, 3011 HP Rotterdam, The Netherlands; 3 Department of Psychology, Education & Child Studies, Faculty of Social & Behavioural Sciences, Erasmus University Rotterdam, Burgemeester Oudlaan 50, 3062 PA Rotterdam, The Netherlands; 4 Department of Obstetrics and Gynaecology, University Medical Centre Utrecht, P.O. Box 85090, 3508 AB Utrecht, The Netherlands; 5 Department of Public Health, Erasmus MC, University Medical Centre, PO Box 2040, 3000 CA Rotterdam, The Netherlands; 6 Department of Biomedical Data Sciences, Leiden University Medical Center, PO Box 9600, 2300 RC Leiden, The Netherlands; 7 Division of Neonatology, Department of Paediatrics, Erasmus MC – Sophia Children’s Hospital, PO Box 2040, 3000 CA Rotterdam, The Netherlands

**Keywords:** Pregnancy, Risk assessment, Risk factors, Epidemiology, Prevention

## Abstract

Social deprivation negatively affects health outcomes but receives little attention in obstetric risk selection. We investigated whether a combination of (1) risk assessment focused on non-medical risk factors, lifestyle factors, and medical risk factors, with (2) subsequent institution of risk-specific care pathways, and (3) multidisciplinary consultation between care providers from the curative and the public health sector reduced adverse pregnancy outcomes among women in selected urban areas in the Netherlands. We conducted a cluster randomised controlled trial in 14 urban municipalities across the Netherlands. Prior to the randomisation, municipalities were ranked and paired according to their expected proportion of pregnant women at risk for adverse outcomes at birth. The primary outcome was delivery of a preterm and/or small for gestational age (SGA) baby, analysed with multilevel mixed-effects logistic regression analysis adjusting for clustering and individual baseline characteristics. A total of 33 community midwife practices and nine hospitals participated throughout the study. Data from 4302 participants was included in the Intention To Treat (ITT) analysis. The intervention had no demonstrable impact on the primary outcome: adjusted odds ratio (aOR) 1.17 (95% CI 0.84–1.63). Among the secondary outcomes, the intervention improved the detection of threatening preterm delivery and fetal growth restriction during pregnancy [aOR 1.27 (95% CI 1.01–1.61)]. Implementation of additional non-medical risk assessment and preventive strategies into general practices is feasible but did not decrease the incidence of preterm and/or SGA birth in the index pregnancy in deprived urban areas.

*Trial registration* Netherlands National Trial Register (NTR-3367).

**Electronic supplementary material:**

The online version of this article (10.1007/s10654-018-0387-7) contains supplementary material, which is available to authorized users.

## Introduction

Social deprivation negatively affects health outcomes. This association is already apparent before birth and extends into early childhood [1–4]. In addition to the negative impact of medical and obstetric risk factors, multiple studies have shown a strong association between non-medical risk factors and adverse pregnancy outcomes. Key examples of such risk factors include low socioeconomic status (SES), living in a deprived neighbourhood, ineffective social integration into society, smoking, and psychosocial stressors [5–8]. The increased prevalence of non-medical risk factors and the accumulation of such factors are responsible for at least part of the overrepresentation of adverse pregnancy outcomes in deprived urban areas within high-income countries [7, 9]. Risk assessment and subsequent implementation of preventive measures in antenatal health care with the aim to reduce adverse pregnancy outcomes should, therefore, take both medical and non-medical risk factors into account. However, current risk selection during pregnancy mainly focuses on medical risks, and integration between the curative and public health sector is scarce [10].

In the Netherlands, obstetric risk selection is based on the ‘List of Obstetric Indications’(LOI), which specifies manifest conditions that define a low, medium, or high-risk pregnancy. These conditions are single medical or obstetric risk factors, that indicate whether a patient’s care during pregnancy or parturition is to be supervised by a community midwife or an obstetrician [11].

The R4U scorecard is a comprehensive risk assessment tool which can, in addition to the LOI, be used by obstetric care providers to identify psychological, social, lifestyle, obstetric and non-obstetric care related factors [12]. The total R4U score is strongly associated with adverse pregnancy outcomes and shows a clear gradient across categories of SES and ethnicity [13].

We conducted a cluster randomised controlled trial (C-RCT) to assess the effectiveness of using the R4U scorecard in conjunction with institution of appropriate care pathways and multidisciplinary consultations, to reduce the incidence of adverse pregnancy outcomes. The study was conducted among pregnant women in selected urban areas in the Netherlands with an overrepresentation of adverse pregnancy outcomes [14–16]. This study is part of the ‘Healthy pregnancy 4 All’ (HP4All) programme, a nationwide study evaluating strategies to improve pregnancy outcomes, in particular among deprived populations [14].

## Methods

### Trial design

We conducted a C-RCT in 14 municipalities in the Netherlands. The Daily Board of the Medical Ethics Committee Erasmus MC approved the study (METC 2012-322). The study protocol was peer-reviewed and published, and was registered at the Netherlands National Trial Register (NTR-3367) [17]. Municipalities were selected based on multiple criteria: (1) size (having more than 70,000 inhabitants), (2) disproportionally high prevalence of risk factors for adverse pregnancy outcomes (high and/or low maternal age, primiparity, non-western ethnicity, and low SES), (3) a high incidence of adverse pregnancy outcomes (delivery of a small for gestational age baby (SGA), preterm delivery, and perinatal mortality [mortality from the 22th week of gestation until 7 days postnatally)], and (4) a higher than average case-fatality rate [14]. The case-fatality rate is the proportion of perinatal mortality amongst neonates with a so-called ‘BIG4’ condition: congenital anomalies, preterm birth, SGA, and/or an Apgar score below seven at five minutes after birth [18]. A more detailed description of the selection process of municipalities has been published before [14]. All community midwife practices and hospitals located in the participating municipalities were invited to participate in the trial.

### Participants

The 14 selected municipalities were divided into ten clusters; five municipalities in the northern part of the Netherlands were merged into one cluster due to the intended formation of a so called ‘obstetric collaborative network’ in that area [17]. An obstetric collaborative network is an inter-professional care system in which community midwives, obstetricians, and maternity care providers share local guidelines and protocols. All women with a singleton pregnancy living in a selected municipality and booking their first antenatal visit at one of the participating community midwife practices or at a participating hospital were eligible for this trial. Exclusion criteria included an obstetric emergency situation or being in labour during the initial visit.

### Intervention

**Fig. 1:**
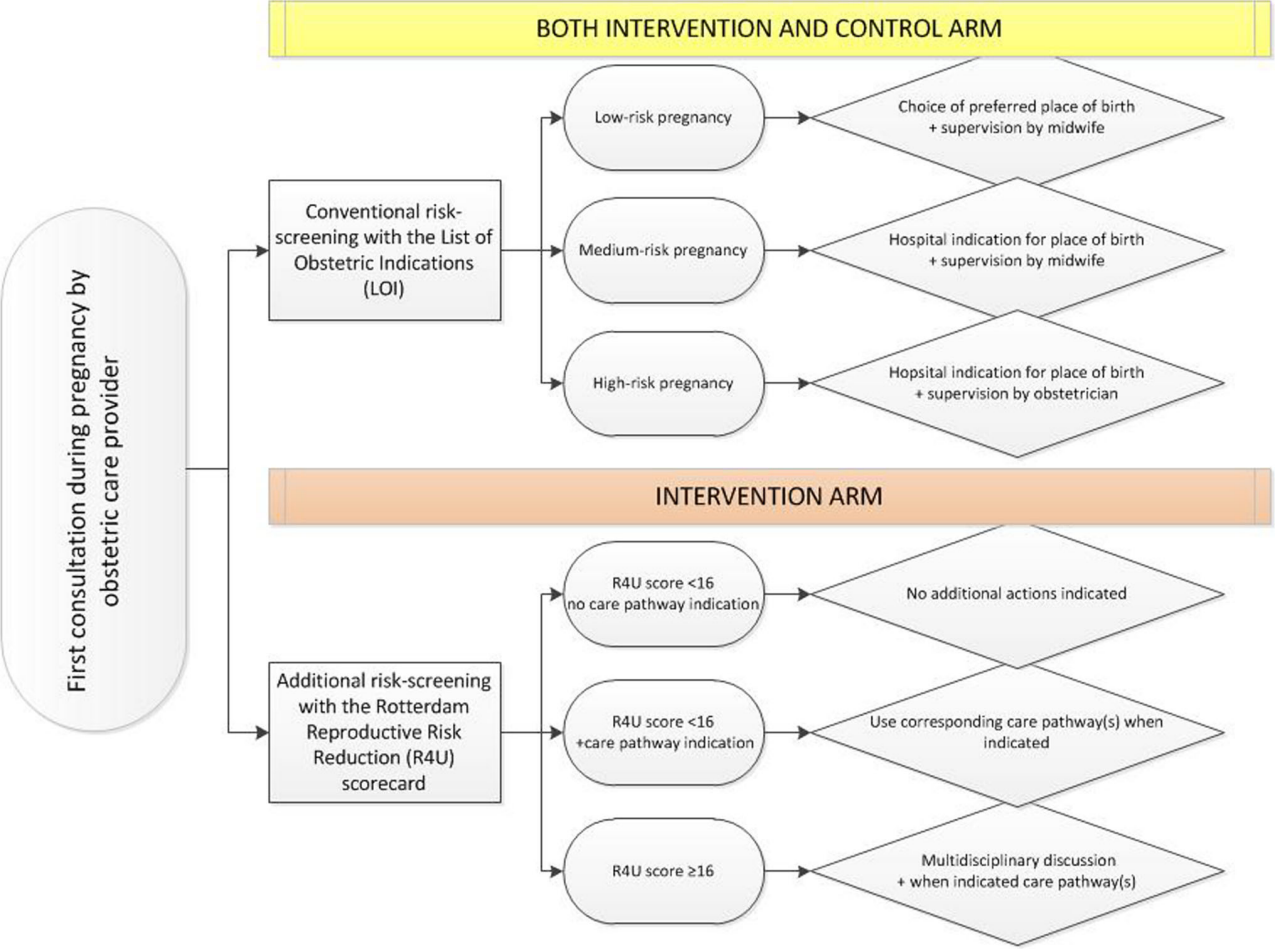
Trial design

In the intervention clusters, participating obstetric care providers used the R4U scorecard as a risk assessment tool at the first antepartum visit. They did so in addition to their conventional risk assessment approach (LOI-based). The R4U scorecard guided coordination of antepartum care through systematic risk assessment for medical and non-medical risk factors for adverse pregnancy outcomes (Online Resource 1). To increase uniformity in questioning within the R4U, a ‘script’ text was formulated for each separate item as a literal text. A positive response indicated presence of the risk factor. Risk factors were selected after a broad literature search and complemented with detailed epidemiological information of prevalence and risk estimates derived from well-documented large birth cohort studies [17]. Risk factors were weighted based on their relative risk for adverse pregnancy outcomes [12]. Scores for individual risk factors were added up to form a total score (range R4U 0-98). A predefined cut-off score was based on data from a pilot study in the Netherlands from 2010 to 2011; a score of 16 points or higher was selected to identify women in the upper 20% of risk scores [19]. In the current study, a cumulative R4U score of ≥ 16 points implied a follow-up action via a case-based discussion in a multidisciplinary setting. In addition, the institution of appropriate individual care pathways was guided by particular single, or a set of multiple, risk factors (Fig. 1).

Case-based multidisciplinary consultations involved community midwives, obstetricians and other healthcare professionals, such as paediatricians or social workers. With this approach optimal linkage was sought between the public health sector and the curative care sector. The aim of the meeting was to agree on a customised antepartum policy for each individual patient [17]. Obstetric care providers were in addition allowed to discuss participants in these discussions to their own discretion, independently of the cumulative R4U score. As a result, participants with relevant individual risk factors could also be discussed when the cumulative score was below 16 (Fig. 1).

Prior to start of the study, 28 templates of care pathways were developed based on the medical and non-medical risk factors incorporated in the R4U. These templates consisted of a set of steps a healthcare professional was advised to take in an attempt to reduce the potential contribution of one or more risk factors to developing an adverse pregnancy outcome. As such, each care pathway could, for example, direct the user to a specific health care provider, or to a health care organisation, public health care organisation, or an office for legal or financial support. When there was existing evidence for interventions to address modifiable risk factors this was used for the contents of the pathway. To enhance the efficiency of the care provided, care pathways are explicit as to which caregiver will be responsible. To facilitate local adaptation of this new way of organising antenatal care, details of the care pathways were discussed during meetings with community midwives, obstetricians, social workers, and a city council representative. In these meetings the templates for the pathways were complemented with the availability of local facilities and health insurance agreements, and refined through direct interaction with relevant local health care providers, and organisations. In addition, the introduction of organized meetings to customize care pathways induced a change in the mutual professional relationship between care providers of different echelons. An example of a care pathway, in this case for psychosocial risk factors, is added in the Online Resource 2.

### Controls

In the control clusters, an existing screening instrument (LOI), which focuses on identification of single, manifest obstetric and medical risks, was used, combined with individual care according to local protocols. The LOI distinguishes between a low, medium, or a high risk pregnancy based on anticipated third trimester and labour complications [11]. Low risk indications allow women to choose the preferred place of delivery (i.e. home birth, birth centre, hospital birth), which is supervised by an independent community midwife. Women with medium risk pregnancies should deliver in a hospital supervised by a midwife, whereas women with high risk pregnancies are supervised by an obstetrician during pregnancy and hospital delivery [11]. If the risk changes from a low or medium risk to a high risk, the woman is referred from primary to secondary or tertiary care, even during labour (Fig. 1).

### Outcomes

Baseline characteristics were collected via a questionnaire that was filled out by participants after the first antepartum visit, generally around ten to 12 weeks of gestational age. The following characteristics were collected: maternal age at inclusion, parity, ethnicity (western versus non-western based on maternal country of birth and classified according to Statistics Netherlands), single motherhood, maternal SES (based on the classification by the Netherlands Institute of Social Research to all postal code areas, and divided into three centile groups: low < 20, medium 20-80, and high > 80) [20], maternal BMI prior to conception (categorised into low < 20, normal 20–29, and high 30 and more), maternal education [(highest completed education categorised into low: primary school, special education, pre-vocational (secondary) education, junior general secondary education; middle: senior general secondary education, pre-university education, senior secondary vocational education; and high: higher professional education, university education)], smoking during pregnancy (yes/no), and risk factors derived from the obstetric history (previous SGA baby, previous preterm delivery).

Perinatal outcome data was collected by a member of the research team 6 weeks after a participant gave birth from medical charts of community midwives and obstetricians.

All predefined outcomes regarding the effectiveness of this intervention pertain to the participant level. The primary study outcome was: delivery of a preterm (i.e. before 37 weeks of gestation) and/or SGA baby (birth weight below the 10th centile adjusted for parity, gestational age, and gender, based on the Dutch reference curves [21]) together referred to as ‘BIG2’.

Secondary outcomes were: the detection of fetal growth restriction during pregnancy (defined as fetal growth below the 10th centile for gestational age) and/or threatening preterm birth during pregnancy (defined by the detection of, and any action taken by an obstetric care provider, after suggestive symptoms of preterm labour), any referral to non-obstetric health care providers during pregnancy used as a proxy for involvement (regardless of referral within the care pathways), any referral to preventive care organisations during pregnancy used as a proxy for involvement (regardless of referral within the care pathways), maternal mortality, unexpected SGA (babies born SGA under supervision of a community midwife), unexpected preterm birth (babies born preterm under supervision of a community midwife), birth asphyxia (an Apgar score below seven at 5 min after birth was used as a proxy), neonatal admission to an intensive care unit, and perinatal mortality (mortality from the 22th week of gestation until 7 days postnatally).

Two secondary outcome measures, as defined in the initial protocol were not analysed, as these outcomes were not considered to be potentially sensitive to a postconceptional intervention. These were: ‘prevalence of general risk factors’ (defined as: pre-existing chronic disease, folic acid use, and medication use) and ‘congenital anomalies’.

Among a sub-cohort we assessed participants’ and health care providers’ satisfaction, and efficacy of implementation of the intervention; these findings are reported elsewhere [22].

### Sample size

Calculation of sample size was based on the presumed effect of the intervention on the primary outcome, and a two-group comparison based on the combined prevalence of preterm birth and SGA in the Netherlands. The intervention was implemented at the municipality level (cluster), while the intervention effect was measured at the participant level. To account for clustering of participants within municipalities, the sample size was multiplied by a Variance Inflation Factor (VIF) of 2.06, calculated with the formula of Donner et al. [23].

In the selected clusters the average incidence of the primary outcome before start of the study (2000–2008) was 16.7%; we hypothesised that the intervention would lead to a decline towards 13% [17]. At an alpha of 0.05 and 80% power, we required 700 participants per cluster, or 3500 participants in each arm. The pre-defined stopping rule was based on the end of the HP4All study period (July 2015).

### Randomisation

We randomised at the level of the clusters. Before the randomisation procedure, municipalities were ranked according to their expected percentage of pregnant women at risk for a ‘BIG2’ outcome at birth. Expected proportions were based on incidence rates from 2000–2008 derived from the Netherlands Perinatal Registry. Municipalities were then paired based on this ranking. The random number generator in R version 2.7.1 was used to assign one of the municipalities in each pair to the intervention arm. The other municipality of that pair was then assigned to the control arm. An independent statistician, who was not involved in executing the study, carried out the randomisation process. The randomisation at cluster level, instead of the randomisation of midwife practices or hospitals, was necessary to avoid contamination as community midwives and obstetricians generally work closely together in obstetric collaborative networks. Obstetric care providers within each cluster were informed and educated with knowledge of the outcome of the randomisation process. Blinding of obstetric care providers was not possible given the nature of the intervention. Allocation concealment of participants was set out by exclusively foreseeing in study information about the situation that was assigned to a specific cluster. As a result, participants were unaware of the randomised design of the study.

### Statistical methods

The impact of the intervention on the primary and secondary outcomes was analysed using multilevel mixed-effects logistic regression analysis (with an assumed random effect for each cluster). Multiple imputation using chained equations was used to account for missing data in baseline characteristics. Both predictor and outcome variables were included to inform the multiple imputation process, forming 15 datasets. Results across the sets were combined using Rubin’s Rules [24]. No interim analysis was performed. Analyses were performed according to the ITT principle. We included the following covariates in our models: age, ethnicity, BMI prior to pregnancy, SES, single motherhood, smoking during pregnancy, and obstetric history (previous SGA baby and/or previous preterm delivery). To account for over-fitting we only analysed secondary outcomes when there were more than ten events in the two groups. Statistical analysis was performed using Stata SE (version 14). Statistical significance was accepted at *p* < 0.05 (two-sided).

### Sensitivity analyses

#### Per protocol analysis

During the trial not all participants in the intervention clusters were screened using the R4U. Therefore, a sensitivity analysis was performed using a per protocol approach to investigate whether this affected the effect estimates.

#### Enrichment of the control clusters

During the study, there was a substantial lag in participant recruitment in the control clusters. Following an ad-hoc study group meeting, a decision was made to ‘enrich’ the control arm with pregnancies included retrospectively from participating practices to a total of 700 participants per cluster. Retrospective pregnancy data was extracted from digital medical charts in participating community midwife practices. Inclusion criteria were identical to the prospective inclusion. Demographic characteristics of the prospectively and retrospectively included participants in the control arm were compared and the potential differences between the groups were explored.

## Results

### Participant flow

**Fig. 2:**
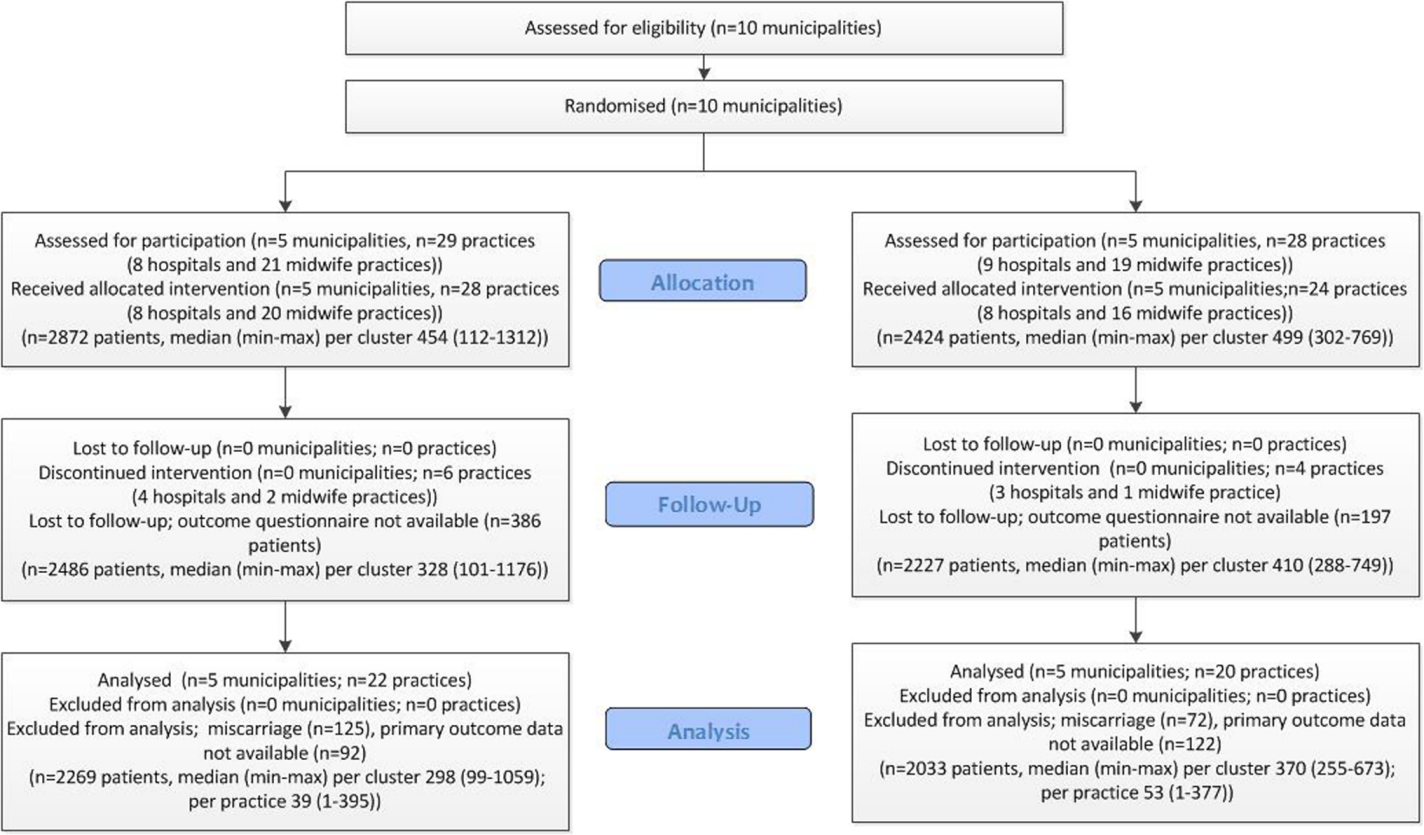
Flow diagram according to CONSORT statement

Five clusters, including eight hospitals and 20 community midwife practices, were included in the intervention arm, and five clusters (eight hospitals and 16 community midwife practices) served as controls (Fig. 2). Complete data regarding baseline characteristics was available for 2486 of 2872 (86.8%) participants in the intervention arm and 2227 of 2424 (91.9%) participants in the control arm (Fig. 2). We excluded participants who had a miscarriage (125 and 72, for the intervention and control group, respectively). Primary outcome data was unavailable for 92 (3.2%) participants in the intervention arm and 122 (5.0%) participants in the control arm (Fig. 2). Accordingly, 4302 participants were included in the ITT analyses.

In the intervention arm, 77.3% of participants were actually screened using the R4U. Of all participants screened 7% had a sum score of 16 or higher, and 50% of participants with an R4U cut-off score above 16 had a registered multidisciplinary consultation.

### Baseline data

Table 1 presents the maternal and pregnancy characteristics at the individual level of all prospectively included participants, by treatment allocation. Online Resource 3, presents the same characteristics at cluster level. Participants in the control arm had a higher income per month, a higher educational level and a higher SES as compared to those in the intervention arm. Participants who did not have data on the primary outcome did not differ importantly from those included in the ITT analysis (Online Resource 4).

**Table 1:** Baseline characteristics of participants

	Intervention (n = 2269)		Control (n = 2033)	
Maternal characteristics				
Age category (years)	N	%	N	%
< 20	16	0.71	17	0.89
20–35	1685	74.33	1431	71.08
> 35	566	24.97	565	28.10
Missing	2	0.09	20	0.98
Ethnic origin				
Western	2020	89.70	1736	85.77
Non-western	232	10.30	288	14.24
Missing	17	0.75	9	0.44
Smoking during pregnancy				
No	1230	81.35	1294	87.79
Yes	282	18.65	182	8.99
Missing	757	33.36	557	27.40
Single mother				
No	1967	95.35	1439	87.14
Yes	96	4.65	46	3.10
Missing	206	9.08	548	26.98
Family income net (euros/month)				
< 1000	116	7.89	106	7.37
1000–1499	233	15.85	176	12.23
1500–1999	203	13.81	160	11.12
2000–2499	228	15.51	195	13.55
2500–2999	239	16.26	204	14.18
> 3000	451	30.68	598	41.56
Missing	799	35.21	594	29.22
Educational level				
Low	199	13.17	179	12.30
Medium	672	44.47	463	31.80
High	640	42.36	814	55.91
Missing	758	33.41	577	28.38
Socioeconomic status				
Low (< P20)	1485	72.79	862	46.59
Medium (P20–P80)	457	22.40	731	39.51
High (> P80)	98	4.80	257	13.89
Missing	229	10.09	183	9.00
BMI at start pregnancy				
BMI < 25	1021	45.20	1098	54.30
BMI 25–35	943	41.74	736	36.40
BMI > 35	295	13.06	188	9.30
Missing	10	0.44	11	0.54
Prior pregnancy characteristics				
Previous SGA baby				
Nulliparous	1079	47.55	985	48.50
No	700	36.12	860	92.18
Yes	159	8.20	73	7.82
Missing	331	14.59	115	10.97
Previous preterm delivery				
Nulliparous	1079	47.55	985	48.50
No	824	41.78	883	93.94
Yes	69	3.50	57	6.06
Missing	297	13.09	108	10.31
Pregnancy characteristics				
Parity				
Nulliparous	1079	47.55	985	48.50
Multiparous	1190	52.45	1048	51.60
Missing	0	0	0	0

Values are expressed as numbers (first) and percentage (second). Percentages of categorised values are percentages of non-missing cases. Missing percentages are percentages of total cases

### Impact of the intervention on primary and secondary outcomes

The combined primary outcome delivery of a preterm and/or a SGA baby (BIG2) occurred in 16.3% of participants in the intervention arm and in 13.2% of participants in the control arm [unadjusted odds ratio OR 1.34 (95% CI 0.92–1.94); Table 2 and Online Resource 5]. The intervention had no demonstrable impact on the primary outcome in multivariable analysis: adjusted odds ratio (aOR) 1.17 (95% CI 0.84–1.63) (Table 3). The intervention improved the detection of threatening preterm delivery and fetal growth restriction during pregnancy compared to the control arm: aOR 1.27 (95%CI 1.01–1.61), but had no significant impact on any other secondary outcomes (Table 3).

**Table 2:** Primary and secondary outcomes at individual level

	Intervention (n = 2269)		Control (n = 2033)	
		%		%
*Primary outcomes*				
BIG2				
Yes	371	16.35	269	13.23
*Secondary outcomes*				
Maternal				
Referral to non-obstetric health care providers				
Yes	523	26.02	568	29.82
Missing	259	11.41	128	6.30
Referral to preventive care organisations				
Yes	129	6.59	74	4.34
Missing	311	13.71	327	16.08
BIG2 detected during pregnancy				
Yes	220	10.59	150	7.69
Missing	192	8.46	83	4.08
Delivery				
Unexpected SGA (delivery of SGA baby in first tier)				
Yes	44	1.95	35	1.73
Missing	7	0.31	11	0.54
Unexpected preterm (preterm delivery in first tier)				
Yes	4	0.18	3	0.15
Missing	10	0.44	13	0.64
Neonatal				
Preterm delivery				
Yes	165	7.28	94	4.63
Small for gestational age				
Yes	229	10.09	186	9.15
Perinatal mortality				
Yes	15	0.67	8	0.40
Missing	35	1.54	10	0.49

Primary and secondary outcomes at individual level, categorised in primary (delivery of a preterm and/or a SGA baby, referred to as ‘BIG2’) and secondary outcomes (maternal, delivery, and neonatal). Values are expressed as numbers (first) and percentage (second). Percentages of categorised values are percentages of non-missing cases. Missing percentages are percentages of total cases

**Table 3:** Impact of intervention on primary and secondary outcomes

	OR (95% CI)	
	Unadjusted	Adjusted
Primary outcomes		
BIG2 (n = 4002)	1.34 (0.92–1.94)	1.17 (0.84–1.63)
Secondary outcomes		
Referral to non-obstetric health care providers (n = 3748)	0.82 (0.54–1.24)	0.79 (0.51–1.23)
Referral to preventive care organisations (n = 3568)	1.17 (0.43–3.21)	0.96 (0.36–2.54)
Fetal growth restriction/preterm birth detected in pregnancy (n = 3830)	1.40 (1.03–1.92)	1.27 (1.01–1.61)
Unexpected SGA (n = 3989)	1.21 (0.69–2.13)	1.28 (0.63–2.62)
Perinatal mortality (n = 3975)	1.70 (0.72–4.02)	1.33 (0.51–3.44)

Numbers are aOR and 95% confidence interval

### Sensitivity analyses

Demographic characteristics differed significantly between the prospectively and retrospectively included participants in the control arm (Online Resource 6). Prospectively included participants were more often of western ethnic origin, had a higher educational level, and a higher SES. Due to this important heterogeneity we decided not to conduct any additional analyses including data from the retrospectively included participants.

#### Per protocol analysis

The effect estimates did not change materially when performing a per protocol analysis as compared to the intention to treat analysis (Table 4).

**Table 4:** Per protocol sensitivity analysis primary and secondary outcome variables

Per protocol analysis	aOR (95% CI)
Primary outcomes	
BIG2 (n = 3564)	1.10 (0.77–1.57)
Secondary outcomes	
Referral to non-obstetric health care providers (n = 3438)	0.79 (0.51–1.22)
Referral to preventive care organisations (n = 3282)	0.95 (0.37–2.42)
Fetal growth restriction/preterm birth detected in pregnancy (n = 3618)	1.32 (0.96–1.80)
Unexpected SGA (n = 3618)	1.04 (0.48–2.29)
Perinatal mortality (n = 3539)	1.29 (0.47–3.53)

Numbers are aOR and 95% confidence interval

## Discussion

By introducing one single tool for additional risk assessment in all tiers of the Dutch obstetric care system we achieved uniformity in risk assessment among 33 community midwife practices and nine hospitals in 14 urban municipalities in the Netherlands [25]. The combination with subsequent institution of care pathways and multidisciplinary consultations further promoted uniformity in a more proactive and preventive approach regarding medical and non-medical risk factors during pregnancy. Hereby the traditional risk assessment during pregnancy, aimed at recognising primarily medical risk factors for complications during labour, shifted towards the first trimester and created a larger window of opportunity for prevention. However in this C-RCT, this combined intervention had no demonstrable impact on preterm and/or SGA birth.

Health inequalities depend on a person’s social, economic, and political environment. These environments are shaped by policies, which makes them amenable to change [26]. Our trial is part of the overall HP4All research programme designed to evaluate the effectiveness of interventions, and their associated preventive strategies, in decreasing health inequalities in pregnancy outcomes [14]. To accomplish implementation of such a programme, interventions should contain a flexible approach that allows for adaptation. Such adaptations stimulate the implementation process and increase sustainability [18]. However, the same flexibility may also have influenced our results. For example, all participating caregivers, including those belonging to the control arm, were educated prior to the start of the program about the importance of non-medical risk factors in relation to adverse pregnancy outcomes. Such adaptations may have resulted in an unintended spill over of intervention effects in the control arm.

This study also has other limitations. Firstly, the intended inclusion of 7000 participants was not achieved within the study’s time frame, and there was a wide variation in sample size among clusters. Secondly, despite the fact that the HP4All programme was set out in the most deprived neighbourhoods of the Netherlands, the participants in this study had an educational level and family income above the national average, suggesting a substantial degree of selection bias. As a result, whereas based on previous research we expected 20% of participants to have an R4U score of 16 or higher, only 7% fulfilled this criterion in the final sample. Thirdly, our results show that not all participants received the intervention as intended. In the intervention arm, 77.4% of all participants were assessed using the R4U scorecard. Of participants with a cut-off score higher than 16, only 50% had a registered multidisciplinary consultation. In addition, the process evaluation of this study, based on Saunders’ 7-step method, showed that only half of the participating municipalities met the criteria for full implementation of the risk assessment program [22]. The combination of not achieving the intended sample size, having fewer participants with a high-risk score according to the pre-defined cut-off, and the above mentioned dilution of the intervention, reduced the power of this study to identify effectiveness.

Fourthly, there were differences in demographic characteristics between participants in the intervention arm and participants in the control arm (Table 1). Participants in the intervention arm had a lower income per month, a lower educational level and a lower SES as compared to those in the control arm. This heterogeneity could be explained by a selection bias, which is a well-recognised phenomenon in C-RCTs [27, 28]. Participants were recruited after the clusters had been randomised. Obstetric care providers had knowledge of whether participants belonged to the intervention or the control arm and this could have affected the types of participants they recruited. Health care providers in the intervention arm may have included more participants with a higher risk for adverse pregnancy outcomes, or in other terms, participants more suitable for ‘active management’. In the control arm this selection likely led to an inclusion of participants with a favourable risk profile. This is further substantiated by the observation that prospectively included participants were more often of western ethnic origin, had a higher educational level, and a higher SES than retrospectively included participants, who were more likely to represent an unbiased sample (Online Resource 6). Although in our analysis we adjusted for known potential confounders, unmeasured confounders could have been imbalanced too and as such may have influenced the results of our analysis.

Despite careful theoretical planning, cluster randomised controlled trials are known to be vulnerable to risk of bias, specifically, bias in selection of participants [27, 29, 30]. Our experience has implications for designing similar trials in the future. The observed inclusion bias in this trial is mostly based on a recruitment bias. Blinding the recruiter of participants for allocation could potentially have diminished this bias. In our trial, participants were recruited by their health care providers, who were also responsible for subsequent pregnancy care, making blinding impossible. This may be addressed by separating participant inclusion from participant care in future studies. Moreover, researchers of C-RCTs may consider conducting an interim analysis, which could potentially have detected the differences in baseline characteristics between the intervention and the control arm. Such an analysis would potentially also have been able to detect the additional issues that eventually caused a dilution of the intervention effect, allowing these to be addressed during the course of the trial.

Despite the above-mentioned limitations, our study shows that implementation of additional non-medical risk assessment and preventive strategies into general practices are feasible. It did, however, not decrease the incidence of adverse perinatal outcomes in the index pregnancy in deprived urban areas.

Extended screening for populations at risk, together with improved collaboration between the curative and public health sector in patient-tailored care, is a start in establishing equity-oriented strategies during pregnancy. However, an intensive research programme as HP4All should ultimately seek to serve pregnant women. Serving in these interventions means detecting those with the greatest health needs, and help them to find the power to direct resources towards those needs. In this perspective, future research in this field should elucidate what empowers pregnant women and which specific resources they need to address their health needs. Effectiveness in this regard, could include value based outcome measures, rather than focusing merely on health outcomes.

## Electronic supplementary material

Below is the link to the electronic supplementary material.

Supplementary material 1 (JPEG 507 kb)

Supplementary material 2 (JPEG 81 kb)

Supplementary material 3 (DOCX 51 kb)

Supplementary material 4 (DOCX 28 kb)

Supplementary material 5 (DOCX 34 kb)

Supplementary material 6 (DOCX 30 kb)
