# Supplementary figures and images for: Antenatal non-medical risk assessment and care pathways to improve pregnancy outcomes: a cluster randomised controlled trial
Source: Eur J Epidemiol. 2018 Mar 31;33(6):579–89. doi: 10.1007/s10654-018-0387-7 (PMC5995981; doi:10.1007/s10654-018-0387-7)

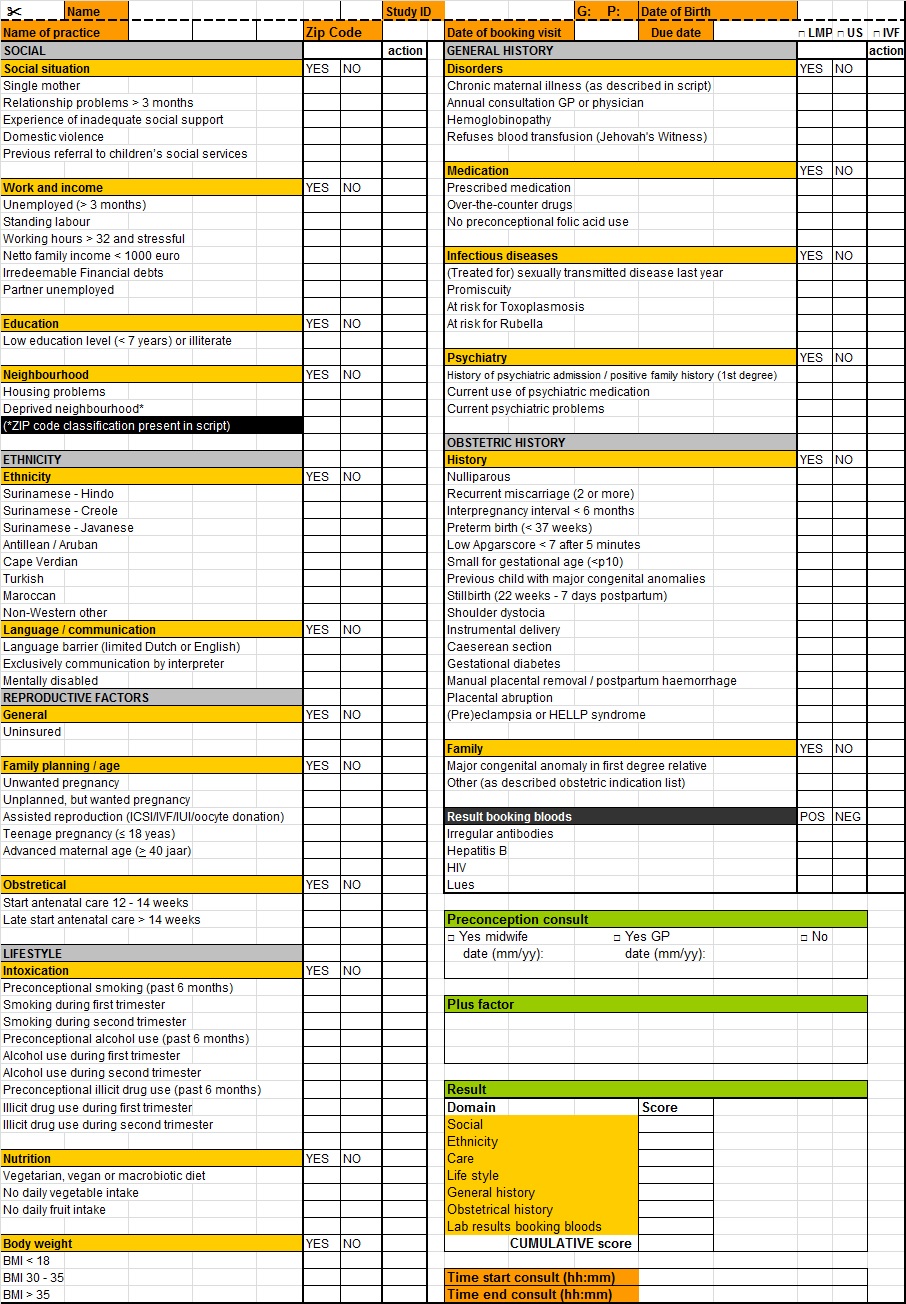

Supplement: Supplementary file 1 — Supplementary material 1 (JPEG 507 kb) [file 10654_2018_387_MOESM1_ESM.jpg]

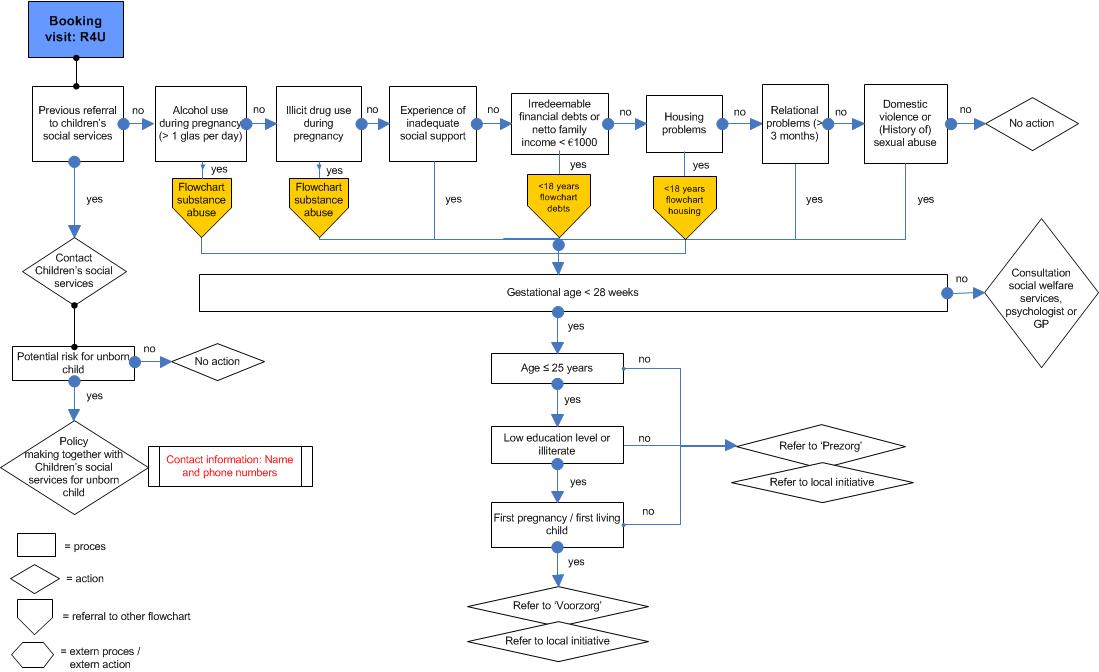

Supplement: Supplementary file 2 — Supplementary material 2 (JPEG 81 kb) [file 10654_2018_387_MOESM2_ESM.jpg]
