# Supplementary material for: Antenatal non-medical risk assessment and care pathways to improve pregnancy outcomes: a cluster randomised controlled trial
Source: Eur J Epidemiol. 2018 Mar 31;33(6):579–89. doi: 10.1007/s10654-018-0387-7 (PMC5995981; doi:10.1007/s10654-018-0387-7)
Supplement: Supplementary file 3 — Supplementary material 3 (DOCX 51 kb) [file 10654_2018_387_MOESM3_ESM.docx]

| **Appendix 3 Baseline characteristics of participants at cluster level** | **Intervention (n=2269)** | | | | | | | | | | | | **Control (n=2033)** | | | | | | | | | | | |
| --- | --- | --- | --- | --- | --- | --- | --- | --- | --- | --- | --- | --- | --- | --- | --- | --- | --- | --- | --- | --- | --- | --- | --- | --- |
| **Cluster level** | 1 (n=298) | | 2 (n=258) | | 3 (n=99) | | 4 (n=555) | | 5 (n=1059) | | **total** | | 6 (n=370) | | 7 (n=457) | | 8 (n=278) | | 9 (n=673) | | 10 (n=255) | | **total** | |
| **Maternal characteristics** | | |  | |  | |  | |  | |  | |  | |  | |  | |  | |  | |  | |
| Age in category | | % |  | % |  | % |  | % |  | % |  | % |  | % |  | % |  | % |  | % |  |  |  | % |
| <20 | 2 | 0.67 | 0 | 0.00 | 1 | 1.01 | 5 | 0.90 | 8 | 0.76 | 16 | 0.71 | 0 | 0.00 | 4 | 0.88 | 1 | 0.36 | 0 | 0.00 | 12 | 5.06 | 17 | 0.85 |
| 20-35 | 191 | 64.09 | 165 | 63.95 | 79 | 79.80 | 415 | 75.05 | 835 | 78.85 | 1685 | 74.33 | 286 | 77.51 | 316 | 69.15 | 206 | 74.37 | 442 | 65.68 | 181 | 76.37 | 1431 | 71.16 |
| >35 | 105 | 35.23 | 93 | 36.05 | 19 | 19.19 | 133 | 24.05 | 216 | 20.40 | 566 | 24.97 | 83 | 22.49 | 137 | 29.98 | 70 | 25.27 | 231 | 34.32 | 44 | 18.57 | 565 | 28.10 |
| Missing | 0 | 0.00 | 0 | 0.00 | 0 | 0.00 | 2 | 0.36 | 0 | 0.00 | 2 | 0.09 | 1 | 0.27 | 0 | 0.00 | 1 | 0.36 | 0 | 0.00 | 18 | 7.06 | 20 | 0.98 |
| Ethnic origin | |  |  |  |  |  |  |  |  |  |  |  |  |  |  |  |  |  |  |  |  |  |  |  |
| Western | 158 | 53.74 | 248 | 97.25 | 91 | 91.92 | 504 | 91.80 | 1019 | 96.59 | 2020 | 89.70 | 328 | 89.62 | 307 | 67.62 | 246 | 88.81 | 617 | 91.82 | 237 | 92.94 | 1736 | 85.86 |
| Non-western | 136 | 46.26 | 7 | 2.75 | 8 | 8.08 | 45 | 8.20 | 36 | 3.41 | 232 | 10.30 | 38 | 10.38 | 147 | 32.38 | 31 | 11.19 | 55 | 8.18 | 17 | 6.67 | 288 | 14.24 |
| Missing | 4 | 1.34 | 3 | 1.16 | 0 | 0.00 | 6 | 1.08 | 4 | 0.38 | 17 | 0.75 | 4 | 1.08 | 3 | 0.66 | 1 | 0.36 | 1 | 0.15 | 0 | 0.00 | 9 | 0.44 |
| Smoking during pregnancy | |  |  |  |  |  |  |  |  |  |  |  |  |  |  |  |  |  |  |  |  |  |  |  |
| No | 169 | 83.66 | 161 | 84.29 | 64 | 90.14 | 299 | 78.89 | 537 | 80.27 | 1230 | 81.35 | 221 | 89.47 | 313 | 85.05 | 130 | 87.84 | 468 | 93.04 | 162 | 77.14 | 1294 | 87.79 |
| Yes | 33 | 16.34 | 30 | 15.71 | 7 | 9.86 | 80 | 21.11 | 132 | 19.73 | 282 | 18.65 | 26 | 10.53 | 55 | 14.95 | 18 | 12.16 | 35 | 6.96 | 48 | 22.86 | 182 | 12.35 |
| Missing | 96 | 32.21 | 67 | 25.97 | 28 | 28.28 | 176 | 31.71 | 390 | 36.83 | 757 | 33.36 | 123 | 33.24 | 89 | 19.47 | 130 | 46.76 | 170 | 25.26 | 45 | 17.65 | 557 | 27.42 |
| Single mother | |  |  |  |  |  |  |  |  |  |  |  |  |  |  |  |  |  |  |  |  |  |  |  |
| No | 219 | 80.22 | 254 | 99.61 | 96 | 96.97 | 508 | 96.95 | 890 | 97.59 | 1967 | 95.35 | 243 | 97.98 | 20 | 5.41 | 146 | 98.65 | 495 | 97.83 | 205 | 96.24 | 1439 | 97.03 |
| Yes | 54 | 19.78 | 1 | 0.39 | 3 | 3.03 | 16 | 3.05 | 22 | 2.41 | 96 | 4.65 | 5 | 2.02 | 350 | 94.59 | 2 | 1.35 | 11 | 2.17 | 8 | 3.76 | 46 | 3.10 |
| Missing | 25 | 8.39 | 3 | 1.16 | 0 | 0.00 | 31 | 5.59 | 147 | 13.88 | 206 | 9.08 | 122 | 32.97 | 87 | 19.04 | 130 | 46.76 | 167 | 24.81 | 42 | 16.47 | 548 | 26.98 |
| Family income net (euros/month) | | |  |  |  |  |  |  |  |  |  |  |  |  |  |  |  |  |  |  |  |  |  |  |
| <1000 | 49 | 17.07 | 4 | 2.13 | 1 | 1.39 | 27 | 7.46 | 35 | 5.30 | 116 | 7.89 | 9 | 3.67 | 44 | 12.57 | 13 | 9.09 | 16 | 3.25 | 24 | 11.54 | 106 | 7.38 |
| 1000-1499 | 52 | 18.12 | 10 | 5.32 | 12 | 16.67 | 43 | 11.88 | 116 | 17.58 | 233 | 15.85 | 20 | 8.16 | 73 | 20.86 | 17 | 11.89 | 30 | 6.09 | 36 | 17.31 | 176 | 12.25 |
| 1500-1999 | 30 | 10.45 | 18 | 9.57 | 9 | 12.50 | 46 | 12.71 | 100 | 15.15 | 203 | 13.81 | 26 | 10.61 | 56 | 16.00 | 20 | 13.99 | 30 | 6.09 | 28 | 13.46 | 160 | 11.13 |
| 2000-2499 | 24 | 8.36 | 24 | 12.77 | 8 | 11.11 | 57 | 15.75 | 115 | 17.42 | 228 | 15.51 | 41 | 16.73 | 51 | 14.57 | 14 | 9.79 | 59 | 11.97 | 30 | 14.42 | 195 | 13.57 |
| 2500-2999 | 9 | 3.14 | 39 | 20.74 | 8 | 11.11 | 59 | 16.30 | 124 | 18.79 | 239 | 16.26 | 43 | 17.55 | 37 | 10.57 | 25 | 17.48 | 65 | 13.18 | 34 | 16.35 | 204 | 14.20 |
| >3000 | 24 | 8.36 | 93 | 49.47 | 34 | 47.22 | 130 | 35.91 | 170 | 25.76 | 451 | 30.68 | 106 | 43.27 | 89 | 25.43 | 54 | 37.76 | 293 | 59.43 | 56 | 26.92 | 598 | 41.61 |
| Missing | 110 | 36.91 | 70 | 27.13 | 27 | 27.27 | 193 | 34.77 | 399 | 37.68 | 799 | 35.21 | 125 | 33.78 | 107 | 23.41 | 135 | 48.56 | 180 | 26.75 | 47 | 18.43 | 594 | 29.25 |
| Educational level | |  |  |  |  |  |  |  |  |  |  |  |  |  |  |  |  |  |  |  |  |  |  |  |
| Low | 50 | 25.13 | 11 | 5.70 | 6 | 8.45 | 50 | 13.26 | 82 | 12.22 | 199 | 13.17 | 25 | 10.12 | 66 | 18.38 | 15 | 10.20 | 21 | 4.25 | 52 | 24.41 | 179 | 12.31 |
| Medium | 86 | 43.22 | 52 | 26.94 | 31 | 43.66 | 160 | 42.44 | 343 | 51.12 | 672 | 44.47 | 92 | 37.25 | 151 | 42.06 | 53 | 36.05 | 64 | 12.96 | 103 | 48.36 | 463 | 31.84 |
| High | 63 | 31.66 | 130 | 67.36 | 34 | 47.89 | 167 | 44.30 | 246 | 36.66 | 640 | 42.36 | 130 | 52.63 | 142 | 39.55 | 79 | 53.74 | 409 | 82.79 | 54 | 25.35 | 814 | 55.98 |
| Missing | 99 | 33.22 | 65 | 25.19 | 28 | 28.28 | 178 | 32.07 | 388 | 36.64 | 758 | 33.41 | 123 | 33.24 | 98 | 21.44 | 131 | 47.12 | 179 | 26.60 | 42 | 16.47 | 577 | 28.41 |
| Social-economic status | |  |  |  |  |  |  |  |  |  |  |  |  |  |  |  |  |  |  |  |  |  |  |  |
| Low (<P20) | 260 | 95.24 | 153 | 59.77 | 66 | 66.67 | 407 | 77.52 | 599 | 67.45 | 1485 | 72.79 | 38 | 12.75 | 227 | 58.66 | 95 | 34.42 | 291 | 44.56 | 211 | 89.41 | 862 | 46.65 |
| Medium (P20 - P80) | 12 | 4.40 | 63 | 24.61 | 27 | 27.27 | 91 | 17.33 | 264 | 29.73 | 457 | 22.40 | 140 | 46.98 | 106 | 27.39 | 155 | 56.16 | 305 | 46.71 | 25 | 10.59 | 731 | 39.56 |
| High (>P80) | 1 | 0.37 | 39 | 15.23 | 6 | 6.06 | 27 | 5.14 | 25 | 2.82 | 98 | 4.80 | 120 | 40.27 | 54 | 13.95 | 26 | 9.42 | 57 | 8.73 | 0 | 0.00 | 257 | 13.91 |
| Missing | 25 | 8.39 | 3 | 1.16 | 0 | 0.00 | 30 | 5.41 | 171 | 16.15 | 229 | 10.09 | 72 | 19.46 | 70 | 15.32 | 2 | 0.72 | 20 | 2.97 | 19 | 7.45 | 183 | 9.01 |
| BMI start pregnancy | |  |  |  |  |  |  |  |  |  |  |  |  |  |  |  |  |  |  |  |  |  |  |  |
| BMI <25 | 110 | 37.29 | 144 | 55.81 | 49 | 49.49 | 272 | 49.10 | 446 | 42.36 | 1021 | 45.20 | 187 | 50.82 | 193 | 42.60 | 153 | 55.04 | 446 | 66.27 | 119 | 46.67 | 1098 | 54.06 |
| BMI 25-35 | 134 | 45.42 | 107 | 41.47 | 45 | 45.45 | 231 | 41.70 | 426 | 40.46 | 943 | 41.74 | 149 | 40.49 | 178 | 39.29 | 112 | 40.29 | 199 | 29.57 | 98 | 38.43 | 736 | 36.24 |
| BMI >35 | 51 | 17.29 | 7 | 2.71 | 5 | 5.05 | 51 | 9.21 | 181 | 17.19 | 295 | 13.06 | 32 | 8.70 | 82 | 18.10 | 13 | 4.68 | 28 | 4.16 | 33 | 12.94 | 188 | 9.26 |
| Missing | 3 | 1.01 | 0 | 0.00 | 0 | 0.00 | 1 | 0.18 | 6 | 0.57 | 10 | 0.44 | 2 | 0.54 | 4 | 0.88 | 0 | 0.00 | 0 | 0.00 | 5 | 1.96 | 11 | 0.54 |
| **Prior pregnancy characteristics** | | |  |  |  |  |  |  |  |  |  |  |  |  |  |  |  |  |  |  |  |  |  |  |
| Previous SGA baby |  |  |  |  |  |  |  |  |  |  |  |  |  |  |  |  |  |  |  |  |  |  |  |  |
| No | 97 | 37.60 | 101 | 41.06 | 42 | 43.30 | 185 | 38.14 | 275 | 32.28 | 700 | 36.12 | 167 | 46.91 | 210 | 51.22 | 116 | 43.45 | 254 | 39.38 | 118 | 49.17 | 860 | 44.89 |
| Yes | 49 | 18.99 | 26 | 10.57 | 4 | 4.12 | 32 | 6.60 | 48 | 5.63 | 159 | 8.20 | 17 | 4.78 | 16 | 3.90 | 13 | 4.87 | 18 | 2.79 | 9 | 3.75 | 73 | 3.81 |
| Missing | 40 | 13.42 | 12 | 4.65 | 2 | 2.02 | 70 | 12.61 | 207 | 19.55 | 331 | 14.59 | 14 | 3.78 | 47 | 10.28 | 11 | 3.96 | 28 | 4.16 | 15 | 5.88 | 115 | 5.66 |
| Previous preterm delivery |  |  |  |  |  |  |  |  |  |  |  |  |  |  |  |  |  |  |  |  |  |  |  |  |
| No | 152 | 53.90 | 121 | 49.39 | 41 | 42.27 | 203 | 41.18 | 307 | 35.91 | 824 | 41.78 | 173 | 48.32 | 216 | 50.94 | 115 | 44.40 | 267 | 41.27 | 112 | 47.26 | 883 | 45.92 |
| Yes | 18 | 6.38 | 5 | 2.04 | 5 | 5.15 | 22 | 4.46 | 19 | 2.22 | 69 | 3.50 | 13 | 3.63 | 24 | 5.66 | 6 | 2.32 | 7 | 1.08 | 7 | 2.95 | 57 | 2.96 |
| Missing | 16 | 5.37 | 13 | 5.04 | 2 | 2.02 | 62 | 11.17 | 204 | 19.26 | 297 | 13.09 | 12 | 3.24 | 33 | 7.22 | 19 | 6.83 | 26 | 3.86 | 18 | 7.06 | 108 | 5.32 |
| **Pregnancy characteristics** | | | | |  |  |  |  |  |  |  |  |  |  |  |  |  |  |  |  |  |  |  |  |
| Parity | |  |  |  |  |  |  |  |  |  |  |  |  |  |  |  |  |  |  |  |  |  |  |  |
| Nulliparous | 112 | 37.58 | 119 | 46.12 | 51 | 51.52 | 268 | 48.29 | 529 | 49.95 | 1079 | 47.55 | 172 | 46.49 | 184 | 40.26 | 138 | 49.64 | 373 | 55.42 | 118 | 46.27 | 985 | 48.50 |
| Multiparous | 186 | 62.42 | 139 | 53.88 | 48 | 48.48 | 287 | 51.71 | 530 | 50.05 | 1190 | 52.45 | 198 | 53.51 | 273 | 59.74 | 140 | 50.36 | 300 | 44.58 | 137 | 53.73 | 1048 | 51.60 |
|  | 0 | 0.00 | 0 | 0.00 | 0 | 0.00 | 0 | 0.00 | 0 | 0.00 | 0 | 0.00 | 0 | 0.00 | 0 | 0.00 | 0 | 0.00 | 0 | 0.00 | 0 | 0.00 | 0 | 0.00 |

Values are expressed as numbers (first) and percentage (second). Percentages of categorised values are percentages of non-missing cases. Missing percentages are percentages of total cases. Prior pregnancy characteristics are presented for multiparous participants.
