# Supplementary material for: Antenatal non-medical risk assessment and care pathways to improve pregnancy outcomes: a cluster randomised controlled trial
Source: Eur J Epidemiol. 2018 Mar 31;33(6):579–89. doi: 10.1007/s10654-018-0387-7 (PMC5995981; doi:10.1007/s10654-018-0387-7)
Supplement: Supplementary file 4 — Supplementary material 4 (DOCX 28 kb) [file 10654_2018_387_MOESM4_ESM.docx]

| **Appendix 4 baseline characteristics of participants with outcome data and participants without outcome data** | **Participants with outcome data (n=4714)** | | **Participants without outcome data (n=604)** | |
| --- | --- | --- | --- | --- |
|  |  | **%** |  | **%** |
| **Maternal characteristics** | | | | |
| Age in category |  |  |  | |
| <20 | 41 | 0,88 | 21 | 3,55 |
| 20-35 | 3399 | 72,74 | 430 | 72,64 |
| >35 | 1233 | 26,39 | 141 | 23,82 |
| Missing | 41 | 0,87 | 12 | 1,99 |
| Ethnic origin |  |  |  |  |
| Western | 4120 | 87,98 | 460 | 77,31 |
| Non-western | 563 | 12,02 | 135 | 22,69 |
| Missing | 31 | 0,66 | 9 | 1,49 |
| Smoking during pregnancy |  |  |  |  |
| No | 2713 | 84,54 | 376 | 87,04 |
| Yes | 496 | 15,46 | 56 | 12,96 |
| Missing | 1505 | 31,93 | 172 | 28,48 |
| Single mother |  |  |  |  |
| No | 3666 | 96,02 | 486 | 93,64 |
| Yes | 152 | 3,98 | 33 | 6,36 |
| Missing | 896 | 19,01 | 85 | 14,07 |
| Family income (euros/month) |  |  |  |  |
| <1000 | 246 | 7,88 | 59 | 14,71 |
| 1000-1499 | 442 | 14,15 | 74 | 18,45 |
| 1500-1999 | 394 | 12,62 | 52 | 12,97 |
| 2000-2499 | 453 | 14,51 | 50 | 12,47 |
| 2500-2999 | 475 | 15,21 | 53 | 13,22 |
| >3000 | 1113 | 35,64 | 113 | 28,18 |
| Missing | 1591 | 33,75 | 203 | 33,61 |
| Educational level |  |  |  |  |
| Low | 419 | 13,14 | 67 | 15,62 |
| Medium | 1219 | 38,24 | 173 | 40,33 |
| High | 1550 | 48,62 | 189 | 44,06 |
| Missing | 1526 | 32,37 | 175 | 28,97 |
| Social-economic status |  |  |  |  |
| Low (<P20) | 2532 | 60,14 | 354 | 62,21 |
| Medium (P20 - P80) | 1289 | 30,62 | 165 | 29,00 |
| High (>P80) | 389 | 9,24 | 50 | 8,79 |
| Missing | 504 | 10,69 | 35 | 5,79 |
| **Pregnancy characteristics** | | | | |
| Parity |  |  |  |  |
| Nulliparous | 2273 | 48,29 | 52 | 44,07 |
| Multiparous | 2434 | 51,71 | 66 | 55,93 |
| Missing | 7 | 0,15 | 486 | 80,46 |
| **Prior pregnancy characteristics** | | | | |
| Previous SGA baby |  |  |  |  |
| No | 1662 | 39,68 | 73 | 34,76 |
| Yes | 251 | 5,99 | 7 | 3,33 |
| Missing | 526 | 11,16 | 394 | 65,23 |
| Previous preterm delivery |  |  |  |  |
| No | 1828 | 43,13 | 69 | 30,13 |
| Yes | 135 | 3,19 | 3 | 1,31 |
| Missing | 476 | 10,10 | 375 | 62,09 |

Values are expressed as numbers (first) and percentage (second). Percentages of categorised values are percentages of non-missing cases. Missing percentages are percentages of total cases. Prior pregnancy characteristics are presented for multiparous participants.
