# Supplementary material for: Antenatal non-medical risk assessment and care pathways to improve pregnancy outcomes: a cluster randomised controlled trial
Source: Eur J Epidemiol. 2018 Mar 31;33(6):579–89. doi: 10.1007/s10654-018-0387-7 (PMC5995981; doi:10.1007/s10654-018-0387-7)
Supplement: Supplementary file 5 — Supplementary material 5 (DOCX 34 kb) [file 10654_2018_387_MOESM5_ESM.docx]

| **Appendix 5 Primary and secondary outcomes at cluster level** | **Intervention (n=2269)** | | | | | | | | | | | | **Control (n=2033)** | | | | | | | | | | | | |
| --- | --- | --- | --- | --- | --- | --- | --- | --- | --- | --- | --- | --- | --- | --- | --- | --- | --- | --- | --- | --- | --- | --- | --- | --- | --- |
| **Cluster level** | 1 (n=298) | | 2 (n=258) | | 3 (n=99) | | 4 (n=555) | | 5 (n=1059) | | **total** | | 6 (n=370) | | 7 (n=457) | | 8 (n=278) | | 9 (n=673) | | 10 (n=255) | | **total** | | |
|  | | % |  | % |  | % |  | % |  | % |  | % |  | % |  | % |  | % |  | % |  |  |  | % | |
| **Primary outcome** | | |  |  |  |  |  |  |  |  |  |  |  |  |  |  |  |  |  |  |  |  |  | |  |
| BIG2 |  |  |  |  |  |  |  |  |  |  |  |  |  |  |  |  |  |  |  |  |  |  |  | |  |
| Yes | 87 | 29.19 | 37 | 14.34 | 15 | 15.15 | 89 | 16.04 | 143 | 13.50 | 371 | 16.35 | 57 | 15.41 | 70 | 15.32 | 33 | 11.87 | 70 | 10.40 | 39 | 15.29 | 269 | | 13.23 |
| **Secondary outcomes** |  |  |  |  |  |  |  |  |  |  |  |  |  |  |  |  |  |  |  |  |  |  |  | |  |
| **Maternal** |  |  |  |  |  |  |  |  |  |  |  |  |  |  |  |  |  |  |  |  |  |  |  | |  |
| Referral to non-obstetric health care providers | | | | |  |  |  |  |  |  |  |  |  |  |  |  |  |  |  |  |  |  |  | |  |
| Yes | 82 | 28.08 | 108 | 43.03 | 19 | 19.19 | 90 | 16.42 | 224 | 27.32 | 523 | 26.02 | 88 | 27.76 | 133 | 29.30 | 84 | 31.34 | 195 | 29.02 | 68 | 35.05 | 568 | | 29.82 |
| Missing | 6 | 2.01 | 7 | 2.71 | 0 | 0.00 | 7 | 1.26 | 239 | 22.57 | 259 | 11.41 | 53 | 14.32 | 3 | 0.66 | 10 | 3.60 | 1 | 0.15 | 61 | 23.92 | 128 | | 6.30 |
| Referral to non-obstetric health care organisations | | | | |  |  |  |  |  |  |  |  |  |  |  |  |  |  |  |  |  |  |  | |  |
| Yes | 47 | 16.15 | 6 | 2.46 | 1 | 1.01 | 25 | 4.58 | 50 | 6.43 | 129 | 6.59 | 14 | 4.75 | 21 | 6.84 | 25 | 9.54 | 8 | 1.19 | 6 | 3.49 | 74 | | 4.34 |
| Missing | 7 | 2.35 | 14 | 5.43 | 0 | 0.00 | 9 | 1.62 | 281 | 26.53 | 311 | 13.71 | 75 | 20.27 | 150 | 32.82 | 16 | 5.76 | 3 | 0.45 | 83 | 32.55 | 327 | | 16.08 |
| BIG2 detected during pregnancy | | | | |  |  |  |  |  |  |  |  |  |  |  |  |  |  |  |  |  |  |  | |  |
| Yes | 40 | 14.18 | 23 | 9.20 | 10 | 10.64 | 46 | 8.91 | 101 | 10.80 | 220 | 10.59 | 17 | 4.83 | 42 | 9.77 | 24 | 9.30 | 41 | 6.18 | 26 | 10.53 | 150 | | 7.69 |
| Missing | 16 | 5.37 | 8 | 3.10 | 5 | 5.05 | 39 | 7.03 | 124 | 11.71 | 192 | 8.46 | 18 | 4.86 | 27 | 5.91 | 20 | 7.19 | 10 | 1.49 | 8 | 3.14 | 83 | | 4.08 |
| **Delivery** |  |  |  |  |  |  |  |  |  |  |  |  |  |  |  |  |  |  |  |  |  |  |  | |  |
| SGA baby in first tier |  |  |  |  |  |  |  |  |  |  |  |  |  |  |  |  |  |  |  |  |  |  |  | |  |
| Yes | 11 | 3.72 | 8 | 3.10 | 0 | 0.00 | 12 | 2.18 | 13 | 1.23 | 44 | 1.95 | 9 | 2.45 | 5 | 1.11 | 3 | 1.08 | 14 | 2.08 | 4 | 1.58 | 35 | | 1.73 |
| Missing | 2 | 0.67 | 0 | 0.00 | 0 | 0.00 | 5 | 0.90 | 0 | 0.00 | 7 | 0.31 | 2 | 0.54 | 6 | 1.31 | 1 | 0.36 | 0 | 0.00 | 2 | 0.78 | 11 | | 0.54 |
| Preterm in first tier | |  |  |  |  |  |  |  |  |  |  |  |  |  |  |  |  |  |  |  |  |  |  | |  |
| Yes | 1 | 0.34 | 0 | 0.00 | 0 | 0.00 | 0 | 0.00 | 3 | 0.28 | 4 | 0.18 | 1 | 0.27 | 1 | 0.22 | 0 | 0.00 | 1 | 0.15 | 0 | 0.00 | 3 | | 0.15 |
| Missing | 2 | 0.67 | 0 | 0.00 | 0 | 0.00 | 6 | 1.08 | 2 | 0.19 | 10 | 0.44 | 2 | 0.54 | 7 | 1.53 | 1 | 0.36 | 0 | 0.00 | 3 | 1.18 | 13 | | 0.64 |
| **Neonatal** | |  |  |  |  |  |  |  |  |  |  |  |  |  |  |  |  |  |  |  |  |  |  | |  |
| Preterm delivery |  |  |  |  |  |  |  |  |  |  |  |  |  |  |  |  |  |  |  |  |  |  |  | |  |
| Yes | 39 | 13.09 | 11 | 4.26 | 9 | 9.09 | 43 | 7.76 | 63 | 5.96 | 165 | 7.28 | 19 | 5.14 | 30 | 6.58 | 14 | 5.04 | 20 | 2.97 | 11 | 4.33 | 94 | | 4.63 |
| Small for gestational age | |  |  |  |  |  |  |  |  |  |  |  |  |  |  |  |  |  |  |  |  |  |  | |  |
| Yes | 58 | 19.46 | 26 | 10.08 | 6 | 6.06 | 49 | 8.83 | 90 | 8.50 | 229 | 10.09 | 40 | 10.81 | 42 | 9.19 | 20 | 7.19 | 54 | 8.02 | 30 | 11.76 | 186 | | 9.15 |
| Perinatal mortality | |  |  |  |  |  |  |  |  |  |  |  |  |  |  |  |  |  |  |  |  |  |  | |  |
| Yes | 3 | 1.03 | 0 | 0.00 | 1 | 1.02 | 5 | 0.91 | 6 | 0.57 | 15 | 0.67 | 3 | 0.81 | 1 | 0.22 | 1 | 0.36 | 2 | 0.30 | 1 | 0.39 | 8 | | 0.40 |
| Missing | 7 | 2.35 | 6 | 2.33 | 1 | 1.01 | 8 | 1.44 | 13 | 1.23 | 35 | 1.54 | 1 | 0.27 | 5 | 1.09 | 2 | 0.72 | 2 | 0.30 | 0 | 0.00 | 10 | | 0.49 |

Supplementary table 3 Primary and secondary outcomes at cluster level, categorised in primary and secondary outcomes (maternal, delivery, and neonatal). Values are expressed as numbers (first) and percentage (second). Percentages of categorised values are percentages of non-missing cases. Missing percentages are percentages of total cases.
