# Supplementary material for: Antenatal non-medical risk assessment and care pathways to improve pregnancy outcomes: a cluster randomised controlled trial
Source: Eur J Epidemiol. 2018 Mar 31;33(6):579–89. doi: 10.1007/s10654-018-0387-7 (PMC5995981; doi:10.1007/s10654-018-0387-7)
Supplement: Supplementary file 6 — Supplementary material 6 (DOCX 30 kb) [file 10654_2018_387_MOESM6_ESM.docx]

**Appendix 6 baseline characteristics of prospective and retrospective participants in the control arm**

|  | **Prospective participants (n=2033)** | | **Retrospective ‘participants’ (n=917)** | |
| --- | --- | --- | --- | --- |
|  | **n** | **%** | **n** | **%** |
| **Maternal characteristics** | | | | |
| Age in category |  |  |  | |
| <20 | 17 | 0.84 | 5 | 0.55 |
| 20-35 | 1431 | 71.09 | 637 | 69.47 |
| >35 | 565 | 28.07 | 275 | 29.99 |
| Missing | 20 | 0.98 | 0 | 0.00 |
| Ethnic origin |  |  |  |  |
| Western | 1736 | 85.77 | 492 | 59.71 |
| Non-western | 288 | 14.23 | 332 | 40.29 |
| Missing | 9 | 0.44 | 93 | 10.14 |
| Smoking during pregnancy |  |  |  |  |
| No | 1294 | 87.67 | 726 | 81.66 |
| Yes | 182 | 12.33 | 163 | 18.34 |
| Missing | 557 | 27.40 | 28 | 3.05 |
| Single mother |  |  |  |  |
| No | 1439 | 96.90 | 852 | 94.77 |
| Yes | 46 | 3.10 | 47 | 5.23 |
| Missing | 548 | 26.96 | 18 | 1.96 |
| Family income (euros/month) |  |  |  |  |
| <1000 | 106 | 7.37 |  |  |
| 1000-1499 | 176 | 12.23 |  |  |
| 1500-1999 | 160 | 11.12 |  |  |
| 2000-2499 | 195 | 13.55 |  |  |
| 2500-2999 | 204 | 14.18 |  |  |
| >3000 | 598 | 41.56 |  |  |
| Missing | 594 | 29.22 |  |  |
| Educational level |  |  |  |  |
| Low | 179 | 12.29 | 53 | 17.61 |
| Medium | 463 | 31.80 | 150 | 49.83 |
| High | 814 | 55.91 | 98 | 32.56 |
| Missing | 577 | 28.38 | 616 | 67.18 |
| Social-economic status |  |  |  |  |
| Low (<P20) | 862 | 46.59 | 307 | 45.48 |
| Medium (P20 - P80) | 731 | 39.51 | 271 | 40.15 |
| High (>P80) | 257 | 13.89 | 97 | 14.37 |
| Missing | 183 | 9.00 | 242 | 26.39 |
| BMI start pregnancy |  |  |  |  |
| BMI <25 | 1098 | 54.30 | 443 | 48.63 |
| BMI 25-35 | 736 | 36.40 | 369 | 40.50 |
| BMI >35 | 188 | 9.30 | 99 | 10.87 |
| Missing | 11 | 0.54 | 6 | 0.65 |
| **Pregnancy characteristics** | | | | |
| Parity |  |  |  |  |
| Nulliparous | 985 | 48.45 | 437 | 47.66 |
| Multiparous | 1048 | 51.55 | 480 | 52.34 |
| Missing | 0 | 0.00 | 0 | 0.00 |
| **Prior pregnancy characteristics** | | | | |
| Previous SGA baby |  |  |  |  |
| No | 860 | 44.84 | 415 | 45.70 |
| Yes | 73 | 3.81 | 56 | 6.17 |
| Missing | 115 | 5.66 | 9 | 0.98 |
| Previous preterm delivery |  |  |  |  |
| No | 883 | 45.07 | 440 | 48.78 |
| Yes | 57 | 2.91 | 25 | 2.77 |
| Missing | 74 | 3.64 | 15 | 1.64 |

Values are expressed as numbers (first) and percentage (second). Percentages of categorised values are percentages of non-missing cases. Missing percentages are percentages of total cases. Prior pregnancy characteristics are presented for multiparous participants.
